# Editorial: Current State of Postural Research - Beyond Automatic Behavior

**DOI:** 10.3389/fneur.2019.01160

**Published:** 2019-10-31

**Authors:** Emily A. Keshner, Joyce Fung

**Affiliations:** ^1^Department of Health and Rehabilitation Sciences, Temple University, Philadelphia, PA, United States; ^2^School of Physical and Occupational Therapy, McGill University, Montreal, QC, Canada

**Keywords:** cognition, attention, technology, Parkinson's Disease, sensorimotor integration

Postural control requires regulating the neural circuitry of musculoskeletal mechanics to maintain and change body spatial orientation to fulfill changing demands specific to the task and the environment. Knowledge progression has closely followed our understanding of the dynamic interplay between organism, task, and environment. Initially, Magnus (1) focused on the reflexes elicited when decerebrate and decorticate animals righted themselves in space. As the science of posturography progressed, minimization of movement reflected through a small center of pressure footprint during quiet stance became the criterion value. A major theoretical shift occurred in the 1970's with development of the dynamic force platform; reactive postural behaviors could be quantified and were found to be adaptive and modifiable. Thus, research began targeting sensory pathways triggering the postural reactions. Simplified mechanics were used to model the multisegmental body as an inverted pendulum with principal motion around the ankle. But recent studies implicate cognitive processing in the organization of postural behaviors. Thus, basic assumptions need to be challenged if posture control research is to continue to evolve. The papers presented in this special issue are evidence of the progress that has been made toward explaining and assessing effective postural control.

## Sensory Information and Postural Control

To discern when multimodal sensory information impacts recovery of upright balance, Le Goic et al. examine subject-specific geometry and inertial parameters. Intrinsic properties of the lower limb, stiffness and damping, were the earliest influences; however, early muscle activity was insufficient to counteract the external forces. The head was the last segment to move; thus, active correction for a fall could not be initiated by vestibular and visual inputs. The authors conclude that proprioception serves as the sole source of information for up to 300 ms following onset of unexpected falls. Rather than improved anticipation with experience, they argue for improved efficiency of reactive behaviors.

Rasman et al. agree that active maintenance of the upright position requires sensorimotor control. Since the relative contribution of each sensory system is determined by its sensor dynamics and the coordinate system to which it links, they argue that active control is required to maintain balance. Thus, sensory contributions to postural control can only be fully interpreted by combining two common protocols: external perturbations and manipulating the balance control loop. Results from a multisegmental robotic balance system that responds to manipulation of sensor dynamics are presented as basic principles underlying standing balance. Alternatively, Peterka et al. provide detailed methods for use and interpretation of a commercially available device to assess central sensorimotor integration. They argue that applying an external balance perturbation clarifies the cause and effect relationships between sensory processing, motor action, and body sway. Data from 40 subjects and prior results from individuals with vestibular deficit are presented to support this conclusion.

Lhomond et al. explore whether re-calibration of sensorimotor mechanisms in the postural control loop occurs during both movement preparation and execution. Facilitating transmission of cutaneous inputs during the planning phase of gait initiation produced increased somatosensory evoked potentials in primary sensorimotor areas; the neural response decreased when standing still. Premotor cortex, specifically supplementary motor area and superior parietal lobe, was concluded to be the putative source of efferent signals that update current body representation by increasing tactile sensitivity. In a review paper, Sienko et al. argue that long-term training with sensory augmentation devices allow time for the nervous system to develop optimal combinations and weights of sensory cues. They report that individuals with vestibular deficit as well as healthy older adults use real-time sensory augmented cues to reduce sway when the initial stance position, support surface, or visual inputs are modified during a static balance task.

## Higher Order Processes and Postural Control

Neuroimaging advances have provided new tools to decipher higher order cortical processing relevant to postural adaptability underlying motor learning and rehabilitation. Using functional magnetic resonance imaging (fMRI) and mental imagery, Patel et al. identified increased activation in dorsolateral prefrontal cortex, superior parietal lobule, inferior occipital gyrus, and lingual gyrus following slip-perturbation training while walking. Imagined slipping increased activity compared to resting state in supplementary motor area, parietal regions, parahippocampal gyrus, and cingulate gyrus. Thus, higher-level processing is required for the timing and sequencing of an effective balance response.

The importance of cortical contributions to postural control is highlighted in the perspective paper by Adkin and Carpenter who have studied the emotional effects of height-induced threat on human postural control. They argue that threat-related postural changes promote a greater physical safety margin while maintaining upright stance. Their critical review of the static balance research literature highlights the need to recognize the potential contributions of psychological and physiological factors on balance deficits associated with age or pathology.

Dakin and Bolton provide a critical review of research methods and progress in the understanding of anticipatory postural behaviors to expose the role of prediction in postural control. Anticipatory behaviors can be facilitated by changes in the state, or set, of the nervous system. They argue that cortical expansion has improved prediction, termed “foresight,” for preparing to interact with the changing environment. Internal models allow for the fine tuning and priming associated with motor affordances, while learning can be implemented in the cortical, basal ganglia, and cerebellar networks.

## Cognition and Attention and Postural Control

Recent research has targeted cognitive and postural interactions to inform about functional behavior in a complex environment. Dual task paradigms are used extensively to reveal the contributions of cognition and attention to postural control, particularly in the aging population. The focused review by Li et al. distinguishes the neural circuits involved in cognitive or motor performance, and asserts that dual task interference should be greatest when the cognitive and motor tasks engage the same neural circuits in keeping with the principle of neural overlap. The literature reveals age-related differences in neural substrates underlying cognition and the degree to which the age-related decline of sensory systems (e.g., vision, hearing) contribute to cognitive load. Findings support focused cognitive training, exercise, and multimodal training of older adults to improve postural and gait outcomes.

Stins and Roerdink assert that maintaining quiet upright stance shifts between postural reflexes and higher (cortical) centers in accordance with the theory of “intermittent control.” This involves a rapid succession of brief periods of postural stability, during which the body dwells relatively motionless in a particular posture, and postural instability, during which the body rapidly transitions to a new stable point. They hypothesize that exerting ballistic control consumes more attention than stiffness control, using variations in reaction time as the index of attention load. Evidence of attentional fluctuations in the control of quiet upright standing is provided by mapping stimulus-response intervals to local COP parameters.

The original research of Chow et al. corroborates the detrimental effect of directing too much conscious attention toward postural control in young but not old subjects. Using an electroencephalography (EEG) method previously identified as an objective indicator of conscious movement control, they assess neural coherence between T3 (verbal-analytical) and Fz (motor-planning) regions of the brain during a challenging balance task with and without directing attention internally to movement production. Increased EEG T3-Fz coherence in conjunction with increased sway path during the internal focus condition is only observed in young subjects. They caution that the observations may not readily translate between populations and are not robust to age-related changes.

By comparing healthy subjects to patients with Radiation-Induced Leukoencephalopathy (RIL), Bargiotas et al. demonstrates that alterations in executive function and attention can lead to postural deficits. Their RIL patients presented isolated dysexecutive syndrome without clinically detectable gait or posture impairment. Postural and visual measurements were made during the ecological task of reading a recipe while cooking. The main finding is that horizontal and vertical eye movements, as well as the average duration of the saccades and fixations, can predict postural deficits in RIL patients. They conclude that increased balance impairment is coupled with a reduced focusing capacity in ecological tasks.

## Impact of Parkinson's Disease on Postural Control

Parkinson's Disease (PD) has often been used as a model for understanding control mechanisms of posture because of the role of basal ganglia in motor planning and intention. Pantall et al. investigated the relationship between cognitive decline and postural dysfunction in individuals with PD. Longitudinal assessments of cognition and postural dynamics were performed. Postural measures were positively correlated with lower cognitive function and increased geriatric depression scores and negatively associated with a qualitative measure of balance confidence. The positive association between motor and non-motor features of PD reflects the potential for shared neural correlates, potentially the subcortical nuclei, between posture and depression.

If the sub-cortical nuclei and pre-supplementary motor areas are complicit in the motor dysfunction observed with PD, then we might expect impairments in motor planning and learning rather than motor production. The ability of individuals with PD to adapt to new task demands was reviewed by Olson et al. Individuals with PD can learn to execute a new motor plan, but have difficulty with rapidly and flexibly switching between plans; thus, their motor plans do not become implicit. It was concluded that individuals with PD may continuously require explicit information and augmented sensory information to create new motor plans. Wright  examined motor adaptation in individuals with PD by manipulating somatosensory input. Support surface incline was changed during prolonged periods of quiet stance. Young healthy controls and aged-matched older adults exhibited long-term aftereffects of this incline, but those with PD did not. These results suggest that the basal ganglia play a role in tonic postural adaptation.

Cortical control for postural demands during walking is particularly challenging for people with PD. Fino et al. examine cognitive-locomotor interference using dual-task paradigms during the gait cycle. Dual tasking interfered with the duration of late swing and from foot contact to weight transference, both of which require higher-order cortical processing for planning and postural adjustments. Fling et al. measured spatial and temporal gait asymmetry in age-matched healthy and individuals with PD while in the levodopa off state. Individuals with PD exhibited significantly more temporal and spatial gait asymmetry than healthy controls, and changes in transcallosal fiber tract integrity of the pre-supplementary motor area (pre-SMA) and S1 was associated with their greater step length asymmetry.

Jacobs et al. propose that the mechanisms underlying low back pain are similar to those of PD. Evidence suggests that both low back pain and PD are associated with impaired proprioceptive function, sensory orientation during standing balance, anticipatory postural adjustments, automatic postural responses, and striatal-cortical function. A review of the data, however, indicated that although both health conditions can be associated with altered striatal-cortical function, the nature of the altered structure or function was different for PD and low back pain.

We learn from this special issue that the human organism is a dynamic system where all components, be they sensory, motor, mechanical, or cognitive, are operating in an interactive and continuous fashion within environmental and task constraints. Although it functions most efficiently as an automatic process, postural control is a motor task that needs to be learned and practiced throughout the lifespan to best serve the motor and cognitive demands presented to the individual performer.

## Author Contributions

EK and JF were co-editors of the special issue and shared all editorial responsibilities and contributions to the writing of this editorial.

### Conflict of Interest

The authors declare that the research was conducted in the absence of any commercial or financial relationships that could be construed as a potential conflict of interest.

## References

[1] Magnus R Animal posture. Proc R Soc Lond Ser B. (1925) 98:339–53.

